# Case Report: Breast cancer in pregnancy with placental metastasis

**DOI:** 10.3389/fonc.2025.1573915

**Published:** 2025-07-28

**Authors:** Yongming Lin, Xinru Xie, Jiayi Wang, Hongbing Chen, Ningning Wan, Jianqing Lin

**Affiliations:** Department of Breast Surgery, The Second Affiliated Hospital of Fujian Medical University, Quanzhou, Fujian, China

**Keywords:** breast cancer, pregnancy, placenta, metastases, mechanism

## Abstract

With the increasing incidence of breast malignant tumours in the population, some rare cases of breast cancer have been reported. We would like to share with you a case of placental metastasis of a malignant tumour of the breast: the patient was a young woman who was diagnosed with a breast cancer at 31 weeks of gestation. After regular follow-up, a healthy baby girl was delivered by caesarean section at 37 weeks of pregnancy. In a subsequent placenta specimen, the pathologist found distant metastasis of breast cancer to the placenta. This is rare in distant metastases of breast cancer. The patient is currently undergoing further anti-tumour therapy. Through reviewing the relevant literature, we have compiled and discussed the mechanisms related to the possible use of placenta in preventing cancer invasion and how breast cancer in pregnancy can be followed up. We hope that sharing this case will help further clinical and experimental research.

## Graphical Abstract

A brief overview of the patient's diagnosis and treatment process.

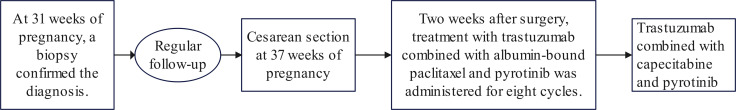

## Introduction

Breast cancer incidence demonstrates a persistent upward trajectory globally (1). Cancer in pregnancy refers to cancer diagnosed during pregnancy or within the first year postpartum. Historically, approximately 1 in 1,000 pregnancies were associated with cancer (2). Pregnancy-associated breast cancer (PABC) was the most common, with an incidence of 39.9 per 100,000 women (3). PABC is often diagnosed at a later stage compared to non-pregnant breast cancer cases (4). Histologically, PABC is more likely to be hormone receptor-negative and to overexpress HER2 (5), which may be associated with adverse outcomes, including an increased risk of metastasis and invasiveness (5–7). Placental metastasis in breast cancer during pregnancy is rare. Certain placental-derived factors, often thought to exert direct anti-tumour effects (8), and the placental microenvironment, which is typically non-supportive of tumour growth (9), may contribute to this rarity. We report a case of a patient diagnosed with breast cancer after pregnancy. After delivery, the placenta was delivered intact, and postoperative pathology revealed placental metastasis of breast cancer. The patient is currently undergoing treatment with the THPy regimen (albumin-bound paclitaxel, trastuzumab, and pyrrolitinib), continue the conventional treatment of trastuzumab combined with capecitabine.

## Case presentation

**Figure 1:**
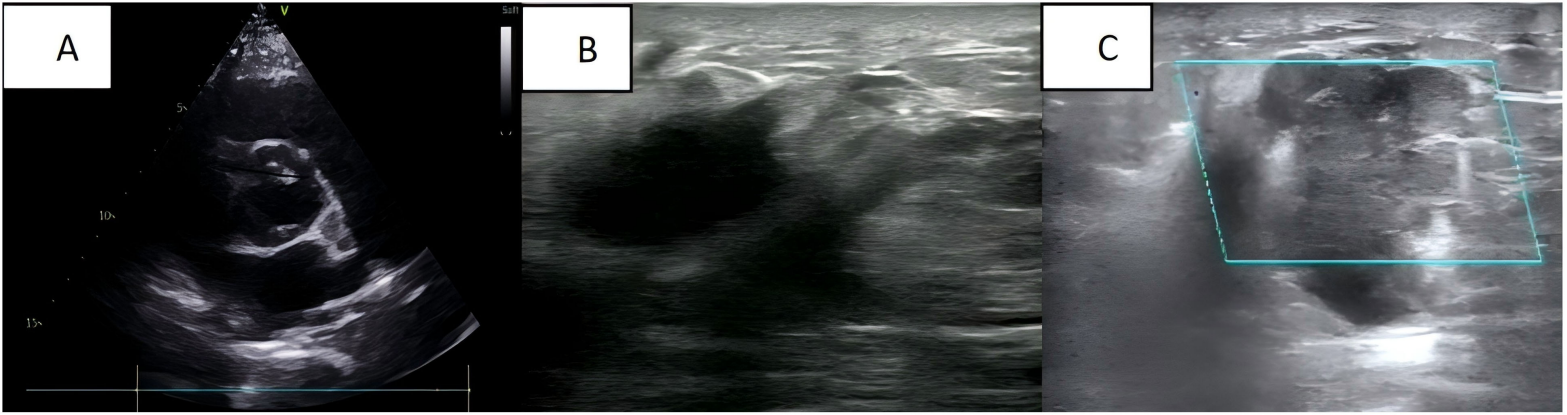
**(A)** Cardiac ultrasound: congenital heart changes are seen; **(B)** Breast ultrasound: a mass is seen in the right breast; **(C)** Axillary ultrasound: an enlarged lymph node is seen in the right axilla.

A 35-year-old Asian woman at 31 weeks of gestation noticed a palpable mass in her right breast with irregular borders and limited mobility, though she reported no specific discomfort. Her medical history included uncorrected congenital heart disease, diagnosed 12 years ago, and was classified as cardiac function class I. She had been followed regularly without further treatment, and her cardiac ultrasound during pregnancy revealed a ventricular septal defect, an arterial ductus arteriosus, and an atrial septal defect (**Figure 1A**). Six years ago, she delivered a healthy baby girl via cesarean section at 37 weeks’ gestation due to her underlying heart condition, and the child’s growth and development have been normal. No additional pregnancies, chronic conditions, or family history of malignancies or genetic disorders were documented. The patient reported occasional alcohol consumption and no tobacco use. At the current visit, a breast ultrasound performed at 35 weeks’ gestation revealed a mass approximately 3.8 × 2.4 × 4.4 cm and multiple hypoechoic nodules in the right breast (**Figure 1B**), raising concern for malignancy and metastasis. Multiple solid lesions in the right axilla, with a maximum size of 2.6 × 2.1 cm, were also noted, with metastatic lymph nodes being suspected (**Figure 1C**).

Ultrasound-guided aspiration biopsy of the breast and axillary lymph nodes was performed. Histological examination confirmed the diagnosis of invasive breast cancer, grade II, with metastasis to the axillary lymph nodes. Immunohistochemical analysis showed weak positivity for oestrogen receptor (ER), weak positivity for progesterone receptor (PR), positivity for HER-2, and a Ki-67 index of 40%.

**Figure 2:**
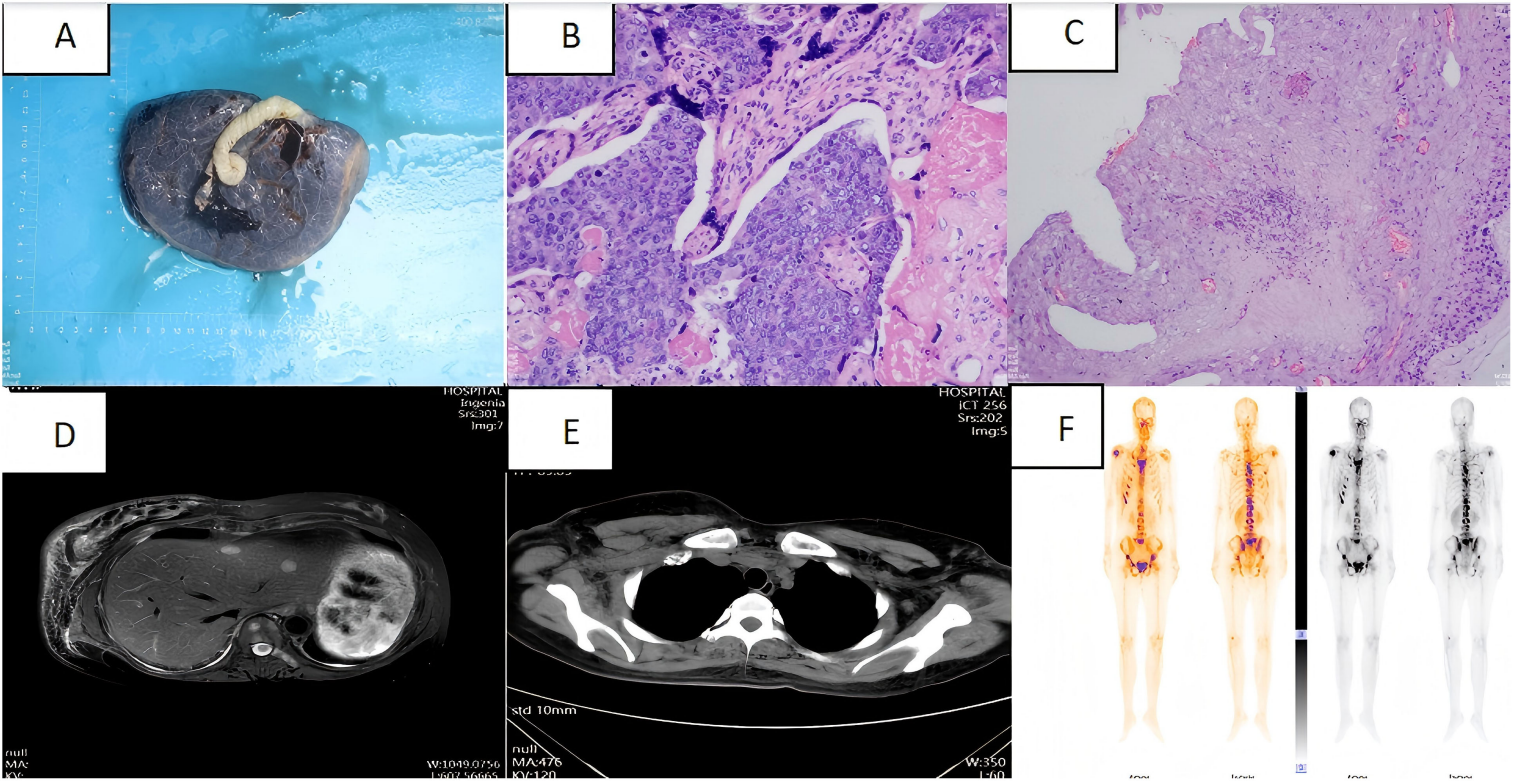
**(A)** Intact placenta after caesarean section; **(B)** Placenta pathology specimen magnified by haematoxylin and eosin staining (high magnification); **(C)** Placenta pathology specimen magnified by haematoxylin and eosin staining (low magnification); **(D)** Abdominal CT: multiple occupations in the liver.; **(E)** Chest CT: multiple enlargement of bilateral axillary, subclavian and mediastinal lymph nodes; **(F)** Whole-body bone imaging: abnormal concentration of bone radioactivity in many places of the body, consider bone metastasis of the tumour.

Following diagnosis, no further antitumor treatment was initiated, and a 2.69 kg baby girl was delivered via cesarean section at 37 weeks of gestation. The procedure included bilateral tubal ligation, pelvic adhesion release, and uterine repair under lumbar and rigid anaesthesia. The newborn had an Apgar score of 10-10-10. The placenta was delivered intact after the surgery (**Figure 2A**). Placental pathology revealed small foci of calcification and infarction within the chorionic villi, along with numerous small foci of heterogeneous epithelioid cell infiltration, which, in combination with morphological and immunohistochemical findings, were consistent with metastasis from breast cancer. Immunohistochemical analysis showed faint positivity for oestrogen receptor (ER) (approximately 10%), progesterone receptor (PR) (approximately 5%), strong positivity for HER-2 (3+), and a Ki-67 proliferation index of approximately 70% (**Figures 2B, C**). Post-delivery imaging revealed liver metastasis and lymph node aggregation in the subclavian region (**Figures 2D, E**). MRI and whole-body bone imaging showed multiple rib and vertebral body metastases (**Figure 2F**). The diagnosis of advanced breast cancer was confirmed, staging the disease as cT2N3aM1, stage IV.

**Figure 3:**
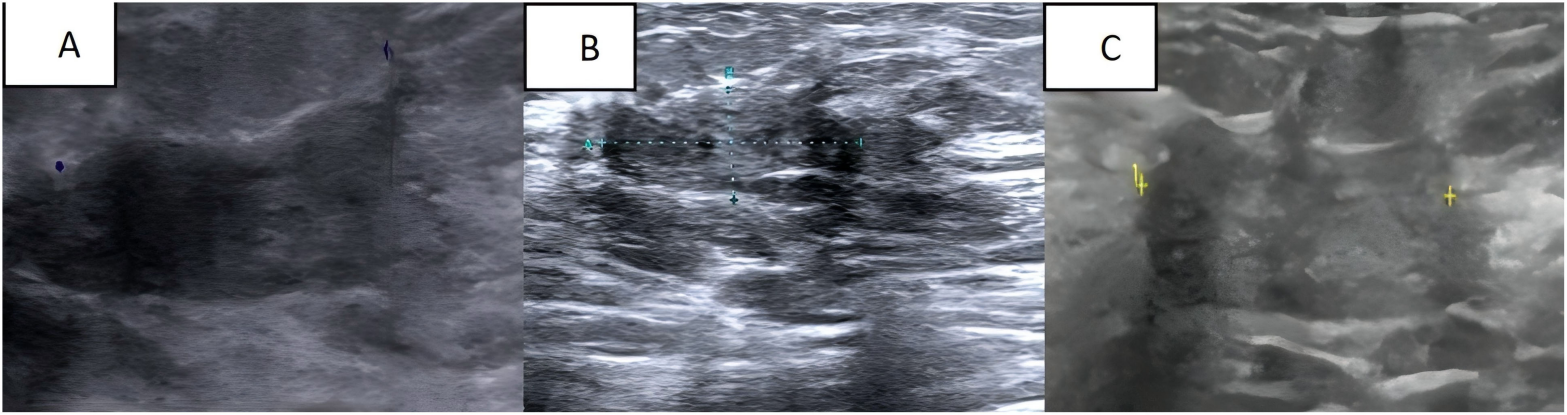
**(A)** Breast colour Doppler ultrasound after 4 cycles of chemical and targeted therapy; **(B)** Breast colour Doppler ultrasound after 8 cycles of chemical and targeted therapy; **(C)** The most recent follow-up breast colour Doppler ultrasound.

The current treatment plan for the patient is 8 cycles of albumin-paclitaxel combined with trastuzumab and pyrotinib, followed by the treatment of pyrotinib combined with capecitabine and trastuzumab. After 4 cycles of chemotherapy, we evaluated the patient’s condition. Colour Doppler ultrasound indicated that the size of the largest tumour focus in the right breast of the patient decreased from 3.8 × 2.4 × 4.4 cm before treatment to 2.5× 2.3 × 0.8 cm (**Figure 3A**), and the therapeutic effect was evaluated as PR(Partial response). After 8 cycles of treatment, the breast colour Doppler ultrasound was reexamined again, showing that the maximum diameter of the tumour was 2.2 × 2.1 × 0.9 cm (**Figure 3B**), and the therapeutic effect was evaluated as SD (Stable disease). At present, the patient is undergoing chemotherapy combined with targeted therapy. After 4 months of medication, the lesion size was evaluated as 2.5 × 2.4 × 1.1 (**Figure 3C**), and the therapeutic effect was evaluated as SD.

During the therapeutic course, serial laboratory assessments (complete blood count and comprehensive metabolic panel) remained within normal limits. Following capecitabine initiation, the patient complained of diarrhoea and improved after symptomatic treatment. In the subsequent follow-up of the infants, we did not find any manifestations of tumour involvement. The growth, development and functional activities of that child was no different from children at the same age. As for the current situation, we temporarily consider that the child patient has not been affected by the maternal disease. After a period of treatment, the current disease progression of the patient is considered stable disease. In the following follow-up, we will continue to pay attention to the conditions of the patient and the child.

## Discussion

This patient was diagnosed with breast cancer during pregnancy with placental metastasis. Placental metastasis of breast cancer is rare. In the literature, there are approximately 17 reported cases of placental metastasis in breast cancer, with no known foetal involvement (10, 11). It is hoped that through the reporting of this case, it can provide assistance for clinical diagnosis and treatment.

About 80 per cent of breast cancers in pregnancy are invasive ductal carcinomas, with the majority presenting with high-grade lesions and lymphovascular infiltration (12). 70% of tumours are oestrogen and progesterone receptor negative compared to non-pregnant patients. Due to its insidious nature of onset, two-thirds of cases present with lymph node metastases at the time of diagnosis, implying a poorer prognosis and a more common likelihood of distant metastases. Meanwhile, due to the particularity of the pregnancy state, some examinations and tests often cannot be completed, which brings obstacles to our diagnosis and disease staging. In previous studies, reproductive factors such as early menarche, late menopause, and late age at first pregnancy have been found to increase the risk of breast cancer, whereas delaying menarche by one year or having an additional child reduces the risk of breast cancer by 5-10% (13, 14). Reproductive factors are closely linked to oestrogen receptor status, and ER-negative tumour cells produce large amounts of growth factors, such as vascular endothelial growth factor and related cytokines, which stimulate various types of cells, including endothelial cells, this is thought to promote the growth of breast cancer cells (15). This may be the reason why breast cancer pathology in pregnancy often shows sex hormone receptor negativity.

Breast tumour growth begins with ductal hyperplasia, the increase in oestrogen levels during pregnancy can lead to ductal hyperplasia and elongation (16). Stimulation of hormone levels may cause breast enlargement during pregnancy with discomfort such as swelling and pain. It can lead to confusion of the enlarged lump with physiological breast enlargement, which can delay treatment and cause problems in our diagnosis. The growth of breast tissue is also stimulated by a surge in luteinising hormone, prolactin. During the first 20 weeks of pregnancy, the immune system is suppressed and this suppression occurs under the influence of hormones (17), which is thought to possibly stimulate active growth of latent breast cancer during this time (18). The response of breast cancer cells to oestrogen and progesterone depends largely on the presence of growth factors, cytokines, the relative concentrations of steroid hormone receptors and their ligands. High concentrations of progesterone induce apoptosis in breast cancer cells and result in the absence of BRCA1 and cell cycle protein A (19). The presence of progesterone in breast cancer cells is also associated with the absence of BRCA1. In the state of pregnancy, the relevant receptors become saturated, functionally active and rapidly transformed, eliminating progesterone receptor-positive mammary epithelial cells by apoptosis under conditions of high circulating progesterone concentrations. It partly explain the protective effect of early pregnancy on breast cancer development and why termination of pregnancy does not significantly improve the prognosis of diagnosed breast cancer (20).

The most common cancer associated with placental metastases is melanoma, which accounts for 31 per cent of placental metastases (21, 22). In a previous review of melanoma placental metastases, 22% of foetuses were affected (23). Affected foetuses have a very poor prognosis, often dying within three months of diagnosis, and melanoma involvement in the foetus is manifested by chorionic villous involvement and is associated with neonatal death. However, cases of placental metastasis of breast cancer involving the foetus have not been reported yet. This might be because the metastatic tumour is was limited to intervillous space (maternal compartment) rather than presenting as villous involvement (11).

Although humans and many other mammals are extremely vulnerable to metastasis of melanoma, melanoma exhibits limited malignancy in mammals such as cattle and horses (24), for example. The incidence of malignancy in mammals has been found to correlate with the extent of placental invasion of the endometrium during pregnancy (25–27). The hypothesis proposes that fibroblasts of the endometrium may evolve to resist invasion by trophoblasts, which also suggest that they may limit the dissemination of tumour cells, and that resistance to trophoblasts correspondingly prevents invasion of tumour cells.

In theory, the blood-rich surface of the placenta is an ideal environment for tumour cell growth (28), but in practice, they are uncommon. In one study, researchers found that implantation of breast cancer cells in the placenta was cleared. During implantation, the placenta can manipulate its surrounding matrix, which may induce the elimination of breast cancer cells (29).

Vertical spread of cancer cells to the placenta or foetus is uncommon in past cases. Most pregnancy-related malignancies metastasise by haematogenous transfer, with lymphatic spread or adjacent invasion being less common routes of metastasis (28). Indeed it is not uncommon to observe malignant cells in the placental vasculature during the metastatic phase of spread of many haematological or solid tumours. Histologically, tumour cells sequestered in the intervillous spaces of the chorionic villi and mixed with maternal blood are observed in all cases of placental metastatic deposits, suggesting haematogenous dissemination of the tumour (30). Maternal and foetal blood circulation is divided into three parts: the trophoblast, the connective tissue of the chorionic villi and the capillary wall. Syncytiotrophoblasts phagocytose maternal cells during the implantation phase, and trophoblast cells may act as a physical barrier in recognising and rejecting foreign maternal antigens expressed by cancer cells. Phagocytosis and destruction of tumour cells by chorionic syncytiotrophoblasts and chorionic trophoblasts have also been reported (31): in a case concerning a leukaemic placental metastasis in a patient at 35 weeks’ gestation, wang et al. observed by electron microscopy that the placenta was morphologically and pathologically different from a normal placenta. They observed phagocytosis of nucleated cells in the chorionic syncytiotrophoblasts. It is well known that this phenomenon usually occurs at the implantation stage and not in the middle of pregnancy. This may also be a mechanism by which the foetal placenta rejects tumour metastasis.

It is well known that angiogenesis is necessary in tumour growth and metastasis (32). It is associated with prognosis and the development of haematogenous metastases. Tumours regulate the generation of new blood vessels through angiogenic and anti-angiogenic growth factors (33). The vascular endothelial growth factor (VEGF) family, which includes several isoforms of VEGF and placental growth factor (PlGF), is produced by alternative splicing of mRNAs (34), which act in autocrine and paracrine pathways (35), and plays a key role in the regulation of angiogenesis (36, 37). The mechanisms are complex and, depending on the source of the tumour tissue, may be controlled by different vascular endothelial growth factor expression pathways or even by the use of other angiogenic factors. Some scholars have noted a lack of angiogenesis in large intervillous metastases. In another study, infarcted areas containing tumour cells were seen in metastatic placenta specimens, and Barr et al. hypothesised that the accumulation of tumour cells on the placenta could lead to a blockage of normal placental metabolism and consequent chorionic villus lysis (38). At the same time, cessation of circulation in the villous capillaries would also help to prevent expansion of the malignant tumour into the foetal region. Difficulty in establishing a vascular network is a factor limiting the expansion of intraplacental metastases. During pregnancy, human chorionic gonadotropin (hCG) induces the production of neovascularisation in various tissues, the placenta being one of them. Its receptor has also been detected in epithelial cells of breast cancer tissues and in breast cancer cell lines. Based on this premise, the normal production of hCG during pregnancy might induce the synthesis of vascular endothelial growth factor, thereby stimulating the development and metastatic potential of breast cancer cells during pregnancy (39).

The mechanism regarding how placental tissue prevents tumour invasion is currently unclear and awaits further theoretical and experimental confirmation, which also provides us with a new research direction, which may provide new ideas and methods for future breast cancer related prevention and treatment.

The rising incidence of gestational malignancies has continued to experts and scholars pay attention to the diagnosis and treatment of related diseases. In studies of breast cancer in pregnancy, it has been found that although many cases of breast cancer in pregnancy are diagnosed at a more advanced stage of the disease, the staged survival rate can be comparable to that of non-pregnant breast cancer if treated properly (40, 41). Newborns born to pregnant women with gestational cancers usually have uncharacteristically poor birth outcomes, studies have shown higher rates of preterm birth and growth restriction compared to healthy mothers (42, 43). It is not clear whether this is an effect of placental vascular disease or placental tumour invasion on the exchange between mother and child. To optimize maternal-foetal outcomes, multidisciplinary team management is essential, with serial prenatal and postnatal surveillance enabling timely clinical assessment. Close follow-up of healthy infants at 6-month intervals over a 2-year period, including physical examination, chest X-ray and liver function tests, is usually recommended (44). Although few cases have been reported, placental pathology can help assess the risk of metastasis in the child. Breastfeeding is discouraged if further systemic therapy is required after delivery to avoid further harm to the child.

## Conclusions

The occurrence of breast cancer during pregnancy poses a challenge to the healthcare professionals involved. We report a case of advanced pregnancy-associated breast cancer (PABC) exhibiting synchronous axillary lymph node metastasis at initial diagnosis, with subsequent confirmation of placental and distant metastases following cesarean delivery. Her child is now growing well and the patient is currently undergoing chemotherapy in combination with targeted therapy, she was aware of and consented to the treatment throughout.

Clinical studies have shown that PABC can be treated with surgery, chemotherapy, and radiation therapy, while endocrine and HER-2 targeted therapies have been found to be of little benefit (2, 45). Terminating pregnancy provides no established survival benefit (46), with most definitive interventions appropriately deferred until delivery. In the recently updated guidelines for the breast cancer during pregnancy, there is no standard treatment for patients with advanced breast cancer (stage III or IV) in the third trimester of pregnancy. Most studies have shown that the 5-year survival rate of these patients is 10%. Further exploration is still needed to obtain better treatment options (47).

The occurrence of placental metastasis of breast cancer in pregnancy is rare, and the mechanism of how the placenta prevents tumour invasion and how the tumour evades the immune system is still unclear and remains to be further investigated.

To elucidate clinicopathological correlations between placental metastasis in pregnancy-associated breast cancer (PABC) and maternal-foetal outcomes, further clinical cases and pathological studies are needed. By reporting this case, we hope to help with subsequent clinical studies.

## Data Availability

The original contributions presented in the study are included in the article/supplementary material. Further inquiries can be directed to the corresponding authors.

## References

[1] Siegel RL Giaquinto AN Jemal A . Cancer statistics, 2024. CA Cancer J Clin. (2024) 74:12–49. doi: 10.3322/caac.21820, PMID: 38230766

[2] de Haan J Verheecke M Van Calsteren K Van Calster B Shmakov RG Mhallem Gziri M . Oncological management and obstetric and neonatal outcomes for women diagnosed with cancer during pregnancy: a 20-year international cohort study of 1170 patients. Lancet Oncol. (2018) 19:337–46. doi: 10.1016/S1470-2045(18)30059-7, PMID: 29395867

[3] Parazzini F Franchi M Tavani A Negri E Peccatori FA . Frequency of pregnancy related cancer: A population based linkage study in lombardy, Italy. Int J Gynecol Cancer. (2017) 27:613–9. doi: 10.1097/IGC.0000000000000904, PMID: 28107260

[4] Javitt MC . Cancer in pregnancy: overview and epidemiology. Abdom Radiol (NY). (2023) 48:1559–63. doi: 10.1007/s00261-022-03633-y, PMID: 35960309

[5] Genin AS Lesieur B Gligorov J Antoine M Selleret L Rouzier R . Pregnancy-associated breast cancers: do they differ from other breast cancers in young women? Breast. (2012) 21:550–5. doi: 10.1016/j.breast.2012.05.002, PMID: 22698618

[6] Loibl S Jackisch C Lederer B Untch M Paepke S Kummel S . Outcome after neoadjuvant chemotherapy in young breast cancer patients: a pooled analysis of individual patient data from eight prospectively randomized controlled trials. Breast Cancer Res Treat. (2015) 152:377–87. doi: 10.1007/s10549-015-3479-z, PMID: 26109347

[7] Baulies S Cusidó M Tresserra F Fargas F Rodríguez I Úbeda B . Biological and pathological features in pregnancy-associated breast cancer: a matched case-control study. Eur J Gynaecol Oncol. (2015) 36:420–3., PMID: 26390695

[8] Froehlich K Schmidt A Heger JI Al-Kawlani B Aberl CA Jeschke U . Breast cancer, placenta and pregnancy. Eur J Cancer. (2019) 115:68–78. doi: 10.1016/j.ejca.2019.03.021, PMID: 31121525

[9] Epstein Shochet G Tartakover Matalon S Drucker L Pomeranz M Fishman A Rashid G . Hormone-dependent placental manipulation of breast cancer cell migration. Hum Reprod. (2012) 27:73–88. doi: 10.1093/humrep/der365, PMID: 22048988

[10] Vetter G Zimmermann F Bruder E Schulzke S Hösli I Vetter M . Aggressive breast cancer during pregnancy with a rare form of metastasis in the maternal placenta. Geburtshilfe Frauenheilkd. (2014) 74:579–82. doi: 10.1055/s-0034-1368181, PMID: 24976641 PMC4069211

[11] Miller TR Rubrecht AE Asirvatham JR Genc MR Shenoy A . Microscopic placental metastasis in triple negative breast carcinoma. Int J Surg Pathol. (2020) 28:521–2. doi: 10.1177/1066896919880964, PMID: 31615307

[12] Rovera F Frattini F Coglitore A Marelli M Rausei S Dionigi G . Breast cancer in pregnancy. Breast J. (2010) 16 Suppl 1:S22–5. doi: 10.1111/j.1524-4741.2010.00998.x, PMID: 21050304

[13] Horn J Vatten L . Reproductive and hormonal risk factors of breast cancer: a historical perspective. Int J Women’s Health. (2017) 9:265–72. doi: 10.2147/ijwh.S129017, PMID: 28490905 PMC5414577

[14] Dall GV Britt KL . Estrogen effects on the mammary gland in early and late life and breast cancer risk. Front Oncol. (2017) 7:110. doi: 10.3389/fonc.2017.00110, PMID: 28603694 PMC5445118

[15] Bando H Toi M Kitada K Koike M . Genes commonly upregulated by hypoxia in human breast cancer cells MCF-7 and MDA-MB-231. BioMed Pharmacother. (2003) 57:333–40. doi: 10.1016/S0753-3322(03)00098-2, PMID: 14568227

[16] Alex A Bhandary E McGuire KP . Anatomy and Physiology of the Breast during Pregnancy and Lactation. Adv Exp Med Biol. (2020) 1252:3–7. doi: 10.1007/978-3-030-41596-9_1, PMID: 32816256

[17] Nelson JH Lu T Hall JE Krown S Nelson JM Fox CW . The effect of trophoblast on immune state of women. Am J Obstetrics Gynecology. (1973) 117:689–99. doi: 10.1016/0002-9378(73)90211-1, PMID: 4742376

[18] Holleb AI Farrow JH . The relation of carcinoma of the breast and pregnancy in 283 patients. Surg Gynecol Obstet. (1962) 115:65–71., PMID: 13908400

[19] Ansquer Y Legrand A Bringuier AF Vadrot N Lardeux B Mandelbrot L . Progesterone induces BRCA1 mRNA decrease, cell cycle alterations and apoptosis in the MCF7 breast cancer cell line. Anticancer Res. (2005) 25:243–8., PMID: 15816544

[20] Gruvberger S Ringnér M Chen Y Panavally S Saal LH Borg A . Estrogen receptor status in breast cancer is associated with remarkably distinct gene expression patterns. Cancer Res. (2001) 61:5979–84., PMID: 11507038

[21] Alexander A Samlowski WE Grossman D Bruggers CS Harris RM Zone JJ . Metastatic melanoma in pregnancy: risk of transplacental metastases in the infant. J Clin Oncol. (2003) 21:2179–86. doi: 10.1200/jco.2003.12.149, PMID: 12775744

[22] Altman JF Lowe L Redman B Esper P Schwartz JL Johnson TM . Placental metastasis of maternal melanoma. J Am Acad Dermatol. (2003) 49:1150–4. doi: 10.1016/s0190-9622(03)00124-5, PMID: 14639405

[23] Lakshminarayana P Danson S Suvarna K Hancock B . Atrial and placental melanoma metastasis: a case report and literature review. J Med Case Rep. (2007) 1:21. doi: 10.1186/1752-1947-1-21, PMID: 17501986 PMC1878489

[24] D’Souza AW Wagner GP . Malignant cancer and invasive placentation: A case for positive pleiotropy between endometrial and Malignancy phenotypes. Evol Med Public Health. (2014) 2014:136–45. doi: 10.1093/emph/eou022, PMID: 25324490 PMC4217742

[25] Wagner GP Kshitiz Levchenko A . 2020: Available data suggest positive relationship between placental invasion and Malignancy. Evol Med Public Health. (2020) 2020:211–4. doi: 10.1093/emph/eoaa024, PMID: 33214901 PMC7658548

[26] Boddy AM Abegglen LM Pessier AP Aktipis A Schiffman JD Maley CC . Lifetime cancer prevalence and life history traits in mammals. Evol Med Public Health. (2020) 2020:187–95. doi: 10.1093/emph/eoaa015/5843791, PMID: 33209304 PMC7652303

[27] Seluanov A Gladyshev VN Vijg J Gorbunova V . Mechanisms of cancer resistance in long-lived mammals. Nat Rev Cancer. (2018) 18:433–41. doi: 10.1038/s41568-018-0004-9, PMID: 29622806 PMC6015544

[28] Jackisch C Louwen F Schwenkhagen A Karbowski B Schmid K Schneider HP . Lung cancer during pregnancy involving the products of conception and a review of the literature. Arch Gynecology Obstetrics. (2003) 268:69–77. doi: 10.1007/s00404-002-0356-x, PMID: 12768292

[29] Epstein Shochet G Drucker L Pomeranz M Fishman A Pasmanik-Chor M Tartakover-Matalon S . First trimester human placenta prevents breast cancer cell attachment to the matrix: The role of extracellular matrix. Mol Carcinog. (2017) 56:62–74. doi: 10.1002/mc.22473, PMID: 26859229

[30] Rothman LA Cohen CJ Astarloa J . Placental and fetal involvement by maternal Malignancy: a report of rectal carcinoma and review of the literature. Am J Obstet Gynecol. (1973) 116:1023–34. doi: 10.1016/s0002-9378(16)33854-6, PMID: 4718214

[31] Wang T Hamann W Hartge R . Structural aspects of a placenta from a case of maternal acute lymphatic leukaemia. Placenta. (1983) 4:185–95. doi: 10.1016/s0143-4004(83)80031-9, PMID: 6576331

[32] Takahashi S . Vascular endothelial growth factor (VEGF), VEGF receptors and their inhibitors for antiangiogenic tumor therapy. Biol Pharm Bull. (2011) 34:1785–8. doi: 10.1248/bpb.34.1785, PMID: 22130231

[33] McColl BK Paavonen K Karnezis T Harris NC Davydova N Rothacker J . Proprotein convertases promote processing of VEGF-D, a critical step for binding the angiogenic receptor VEGFR-2. FASEB J. (2007) 21:1088–98. doi: 10.1096/fj.06-7060com, PMID: 17242158

[34] Houck KA Ferrara N Winer J Cachianes G Li B Leung DW . The vascular endothelial growth factor family: identification of a fourth molecular species and characterization of alternative splicing of RNA. Mol Endocrinol. (1991) 5:1806–14. doi: 10.1210/mend-5-12-1806, PMID: 1791831

[35] Tischer E Mitchell R Hartman T Silva M Gospodarowicz D Fiddes JC . The human gene for vascular endothelial growth factor. Multiple protein forms are encoded through alternative exon splicing. J Biol Chem. (1991) 266:11947–54. doi: 10.1016/S0021-9258(18)99049-6, PMID: 1711045

[36] Shibuya M Claesson-Welsh L . Signal transduction by VEGF receptors in regulation of angiogenesis and lymphangiogenesis. Exp Cell Res. (2006) 312:549–60. doi: 10.1016/j.yexcr.2005.11.012, PMID: 16336962

[37] Ferrara N Davis-Smyth T . The biology of vascular endothelial growth factor. Endocr Rev. (1997) 18:4–25. doi: 10.1210/edrv.18.1.0287, PMID: 9034784

[38] Barr JS . Placental metastases from a bronchial carcinoma. J Obstet Gynaecol Br Emp. (1953) 60:895–7. doi: 10.1111/j.1471-0528.1953.tb07292.x, PMID: 13131128

[39] Michel RM Aguilar JL Arrieta O . Human chorionic gonadotropin as an angiogenic factor in breast cancer during pregnancy. Med Hypotheses. (2007) 68:1035–40. doi: 10.1016/j.mehy.2006.05.072, PMID: 17112680

[40] Amant F von Minckwitz G Han SN Bontenbal M Ring AE Giermek J . Prognosis of women with primary breast cancer diagnosed during pregnancy: results from an international collaborative study. J Clin Oncol. (2013) 31:2532–9. doi: 10.1200/JCO.2012.45.6335, PMID: 23610117

[41] Cardonick E Dougherty R Grana G Gilmandyar D Ghaffar S Usmani A . Breast cancer during pregnancy maternal and fetal outcomes. Cancer J. (2010) 16:76–82. doi: 10.1097/PPO.0b013e3181ce46f9, PMID: 20164696

[42] Van Calsteren K Heyns L De Smet F Van Eycken L Gziri MM Van Gemert W . Cancer during pregnancy: an analysis of 215 patients emphasizing the obstetrical and the neonatal outcomes. J Clin Oncol. (2010) 28:683–9. doi: 10.1200/JCO.2009.23.2801, PMID: 19841323

[43] Amant F Vandenbroucke T Verheecke M Fumagalli M Halaska MJ Boere I . Pediatric Outcome after Maternal Cancer Diagnosed during Pregnancy. N Engl J Med. (2015) 373:1824–34. doi: 10.1056/NEJMoa1508913, PMID: 26415085

[44] Pavlidis N Pentheroudakis G . Metastatic involvement of placenta and foetus in pregnant women with cancer. Recent Results Cancer Res. (2008) 178:183–94. doi: 10.1007/978-3-540-71274-9_16, PMID: 18080453

[45] Boere I Lok C Poortmans P Koppert L Painter R Vd Heuvel-Eibrink MM . Breast cancer during pregnancy: epidemiology, phenotypes, presentation during pregnancy and therapeutic modalities. Best Pract Res Clin Obstet Gynaecol. (2022) 82:46–59. doi: 10.1016/j.bpobgyn.2022.05.001, PMID: 35644793

[46] Cardonick E . Pregnancy-associated breast cancer: optimal treatment options. Int J Womens Health. (2014) 6:935–43. doi: 10.2147/IJWH.S52381, PMID: 25395871 PMC4226455

[47] Board PDQATE . Breast cancer treatment during pregnancy (PDQ^®^): health professional version. In: PDQ cancer information summaries. National Cancer Institute (US, Bethesda (MD (2002).

